# AI-Powered Microscopic Diagnostic Techniques for *Candida albicans* Detection: A Systematic Review 

**DOI:** 10.30476/dentjods.2025.104629.2540

**Published:** 2026-03-01

**Authors:** Reyhaneh Shoorgashti, Farnaz Jafari, Simin Lesan

**Affiliations:** 1 Dept. of Oral and Maxillofacial Medicine, School of Dentistry, Islamic Azad University of Medical Sciences, Tehran, Iran.; 2 Oral and Dental Diseases Research Center, Kerman University of Medical Sciences, Kerman, Iran.

**Keywords:** Artificial Intelligence, Candida albicans, Deep Learning, Machine Learning

## Abstract

**Background:**

Artificial intelligence (AI) powered technologies can help detect *Candida albicans* (*C. albicans*) infections, which are a public health challenge due to increasing incidence rates and conventional therapy resistance.

**Purpose:**

This review explores recent advancements, methodologies, and clinical implications in the AI-driven microscopic detection of *C. albicans*.

**Materials and Method:**

A literature search was conducted across multiple databases, including PubMed, Scopus, Embase, Web of Science, and Google Scholar. Following a thorough review of the retrieved articles, 7 studies were selected for inclusion in this review.

**Results:**

This review analyzed 7 studies that employed AI and machine learning (ML) to detect the presence of *C. albicans*. The most commonly used dataset for detecting *C. albicans* through AI was microscopic images. Two studies employed time-lapse microscopy, and another study used the microorganism's smell fingerprint or volatile organic compounds with an impressive accuracy of 97.70%. The accuracy of detecting *C. albicans* through AI using microscopic images ranged from 63% to 100% depending on the model used.

**Conclusion:**

AI can improve the detection of *C. albicans* infections. It can enhance the accuracy, speed, and efficiency of detection, providing clinicians with invaluable support in identifying infections earlier, optimizing treatment strategies, and ultimately improving patient outcomes.

## Introduction

Fungal infections pose a serious challenge in intensive care units (ICUs), significantly increasing patient morbidity and mortality. Among these, candidiasis, primarily caused by *Candida albicans (C. albicans)*, is the most prevalent form of invasive fungal infection [1-4]. *C. albicans* is a commensal yeast, commonly found in the human microbiota, particularly on the mucosal surfaces of the gastrointestinal and genitourinary tracts [5-9].

While typically harmless in healthy individuals, *C. albicans* can become pathogenic in those with compromised immune systems or underlying health conditions [6, 9-11]. The clinical manifestations of candidiasis range from mild mucosal infections- such as oral thrush and vaginal yeast infections- to severe, potentially life-threatening systemic infections like candidemia [7, 10, 12-13].

Early and accurate diagnosis of candidiasis is critical, given the limited range of effective antifungal treatments and the necessity for prompt therapeutic intervention. However, conventional diagnostic methods often suffer from delayed turnaround times, which hinder timely treatment and adversely affect patient outcomes [14].

Currently, the gold standard for diagnosing invasive candidiasis includes histopathological analysis, positive cultures from sterile sites, microscopic identification of yeast in tissue specimens, and detection in blood cultures [15]. Although non-culture-based methods- such as beta-D-glucan assays, germ tube antibody detection, nucleic acid amplification tests, and T2 Biosystems-based diagnostics- are available, they are typically expensive, require specialized expertise, and are often inaccessible in resource-limited settings. These methods also involve processing times of 72-96 hours, with antifungal susceptibility testing requiring an additional 48–72 hours, further delaying treatment and increasing mortality [15-17].

Given these challenges, there is an urgent need for innovative, rapid, and practical diagnostic approaches for detecting *Candida* infections.

Artificial intelligence (AI) offers promising solutions in this context. By leveraging large datasets, AI systems can detect complex patterns and features, enabling advances across various domains, including healthcare [18-24]. Originally conceptualized in the 1940s, AI refers to the ability of machines to perform tasks that typically require human intelligence [18-19, 25]. A key subset of AI is machine learning (ML), which allows systems to learn patterns from data through statistical and probabilistic modeling without being explicitly programmed [19, 24, 26]. Within ML, deep learning (DL) uses neural networks with multiple hidden layers to capture high-level abstractions in data, enabling more accurate predictions and classifications [23, 27-28].

In infectious disease management, AI has demonstrated substantial potential in the detection, classification, and prediction of pathogens [29-35]. By integrating data from genomic, proteomic, and clinical sources, AI models can uncover subtle indicators of *C. albicans* presence, facilitating rapid and precise diagnosis and supporting timely clinical decision-making [1, 32, 34-37]. Additionally, AI has transformed drug discovery by accelerating the identification and optimization of new therapeutic agents through sophisticated data analysis techniques [38-43].

This systematic review aims to explore the evolving role of AI in the detection and management of *C. albicans* infections, highlighting recent technological advancements and their potential to reshape current diagnostic practices.

## Materials and Method

The systematic review's reporting adheres to the Preferred Reporting Items for Systematic Reviews and Meta-Analyses, extension for Diagnostic Test Accuracy Studies (PRISMA-DTA) guideline [44]. Furthermore, the protocol for this review was registered at PROSPERO under the registration number CRD42024541235. This ensures transparency and rigor in the review process, aligning with established standards in the field of diagnostic test accuracy studies.

The study evaluated relevant research based on the PICO question exploring whether AI can enhance the diagnostic accuracy of *C. albicans* compared to the established reference standard. We included studies that met the following criteria:

Population: Studies utilized AI-powered technologies to detect and classify *C. albicans*;

Intervention and comparison: AI-driven detection and classification of *C. albicans* compared to reference standard tests;

Outcomes: Accuracy, sensitivity or recall, precision, specificity, and F1-score.

In case of more than one result, the best performance was reported.

Conference abstracts, case reports, and review literature were excluded from the study.

A thorough search of relevant literature was performed in several databases including PubMed, Scopus, Embase, Web of Science, and the Google Scholar search engine, up to February 2024.

The search was limited to solely journal publications, with no constraints on language or publication year. Customized keywords and search queries were utilized for each database (Table 1). Besides the electronic search, a manual search was conducted among the listed included studies to uncover any potentially missed articles (Table 1).

Citation management was conducted using Endnote X9 (Clarivate, Philadelphia, PA, USA). Initially, a search yielded 2,245 studies. After eliminating duplicate articles (n= 357) using the “Find Duplicates” option in

**Table 1:** The strategy for Boolean search

| Database | Keywords | Results |
|---|---|---|
| PubMed | ("candida albicans"[MeSH Terms] OR ("candida"[All Fields] AND "albicans"[All Fields]) OR "candida albicans"[All Fields]) AND ("artificial intelligence"[MeSH Terms] OR ("artificial"[All Fields] AND "intelligence"[All Fields]) OR "artificial intelligence"[All Fields] OR ("machine learning"[MeSH Terms] OR ("machine"[All Fields] AND "learning"[All Fields]) OR "machine learning"[All Fields]) OR ("deep learning"[MeSH Terms] OR ("deep"[All Fields] AND "learning"[All Fields]) OR "deep learning"[All Fields])) | 76 |
| Google Scholar | allintitle: ("candida albicans") AND ("artificial intelligence" OR "machine learning" OR "deep learning") | 3 |
| Embase | ('candida albicans'/exp OR 'candida albicans') AND ('artificial intelligence'/exp OR 'artificial intelligence' OR 'machine learning'/exp OR 'machine learning' OR 'deep learning'/exp OR 'deep learning') | 1,431 |
| Scopus | TITLE-ABS-KEY (( "candida albicans") AND ("artificial intelligence" OR "machine learning" OR "deep learning")) | 123 |
| ScienceDirect | ("candida albicans") AND ("artificial intelligence" OR "machine learning" OR "deep learning") | 612 |

EndNote X9 followed by manual verification by R.S., 2,033 articles remained for further evaluation. Upon evaluating the titles and abstracts of these articles and discarding studies deemed irrelevant independently by two evaluators (R.S. and F.J.) (n= 2,011), 22 studies were selected for a detailed full-text assessment. Any disagreements were resolved through consensus involving a third investigator (S.L.). Ultimately, 7 studies met the criteria for inclusion in this review.

The data extraction procedure entailed independent extraction of data from the full text of the included articles by two reviewers (R.S. and F.J.), resulting in a substantial inter-rater agreement rate of 96%. A third reviewer (S.L.) also reviewed the extracted data which encompassed the following information: the primary author's name, publication year, study object, Candida species analyzed, the data set used for training, validation, and testing of the model, inclusion and exclusion criteria for each study, any pre-processing or augmentation techniques used, the type of model employed, its task and performance.

Two reviewers (R.S. and F.J.) independently assessed the risk of bias in each study using the Quality Assessment of Diagnostic Accuracy Studies-2 (QUADAS-2) tool [45]. Any disagreements were resolved through consensus with a third investigator (S.L.).

## Results

### Study Selection and Characteristics

The study selection process is outlined in Figure 1 using the PRISMA flow diagram. After screening and applying eligibility criteria, 7 studies were included in this systematic review. These studies were published between 2021 and 2024, with a noticeable increase in pub-

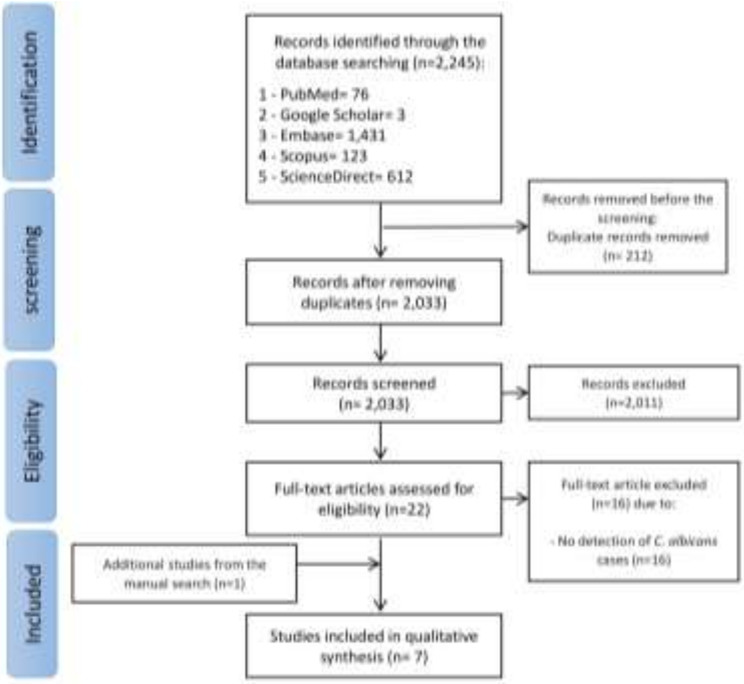

**Figure 1:** The flowchart of the search in this review lications in 2024 (Figure 2). A summary of their characteristics is presented in Table 2 (Figures 1-2, Table 2).

#### Data Modalities

The included studies utilized 3 primary data modalities for AI-based detection of *C. albicans* including (1) microscopic images (n=5) [14, 37, 46-48]; (2) time-lapse microscopy videos (n=2) [46, 49]; and (3) volatile organic compounds (VOC) (microorganisms' smell fingerprint) (n=1) [1].

Several studies included *C. albicans* alongside other species for multi-class classification, while others focused solely on *C. albicans* detection.

#### Performance of Microscopic Image-Based AI Models

Five studies employed microscopic images to train and test AI models for classifying *C. albicans* [14, 37, 46-48]. Analysis of the image dataset revealed varying accuracy ranges of AI-based models, ranging from 63% to 100%. Sensitivity ranged from 56% to 88.5%, precision from 62% to 100%, and F1 score from 59% to 88%.

These studies used convolutional neural networks

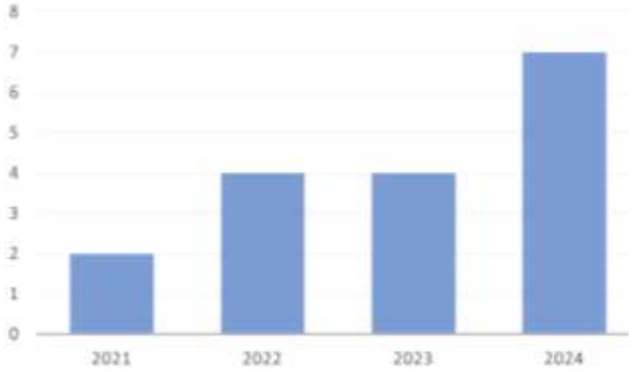

**Figure 2:** Cumulative total number of published studies

**Table 2:** Summary of the findings of included studies

| Author/ Year | Objective | Type of Species | Data Set (Training, Validation, Testing) | Eligibility criteria (if available) Labeling procedure | Machine Learning Task | Pre-processing | Type of Dataset | Model | Performance measured | Outcomes |
|---|---|---|---|---|---|---|---|---|---|---|
| Bastos 2024 [1] | Identification of *Candida spp.* | *C. albicans, C. glabrata, C. haemulonii, C. kodamaea ohmeri, C. krusei, C. parapsilosis* | 397 (238/ 79/ 80 cycles) | Laboratory labeling and cultivating in Petri dishes | Classification | *NA* | Time series data by Volatile organic compounds | KNN, Inception time, TSFC, STC, RISE, ROCKET, BOSS, HIVE COTE 1, HIVE COTE 2 | Accuracy<br>Sensitivity<br>Specificity<br>Precision<br>F1-score | 97.46 %<br>97.81 %<br>99.51 %<br>97.54 %<br>97.60 % |
| Sarkar 2024 [46] | Enhancing the classification accuracy of the *Candida spp.* using neutrophil morphodynamics | *C. albicans, C. glabrata* | Testing: 144 videos 1152 videos in 9 cycles (80%/ 20%/-) | Assigning each image or video a label indicating the infection scenario | Classification | Reduce noises, blending blurred images with Original Images, enhanced contrasts, unsharp masking, clipping Pixel Values, resizing | Microscopic images Time-lapse microscopy videos | CNN (ResNet50, InceptionV3, Xception, EfficientNetB5) RNN (GRU) Transformers AMIT | Accuracy<br>Recall<br>Precision<br>F1-score | 100%<br>76%<br>80%<br>78% |
| Shankarnarayan 2024 [14] | Identification of *Candida spp.* | *C. albicans, C. auris, C. glabrata, C. haemulonii* | 19000 images (12800/ 3200/ 6200) | Problematic images were excluded (blurred, non-focused images, low-cell counts images) | Classification | Resize, rescale the pixel value, augmentation (random rotation, translation, flip, zoom) | Microscopic images | custom CNN VGG16, ResNet50, InceptionV3, EfficientNetB0, EfficientNetB7 | Accuracy<br>Sensitivity<br>Specificity<br>Precision<br>F1-score<br>AUC | 74.6%<br>77.1%<br>92.4%<br>77.9%<br>77.14 %<br>91.9% |
| Belyaev 2022 [49] | Analyzing neutrophils response when encountering fungal pathogens | *C. albicans, C. glabrata* | *NA* | *NA* | Classification | Automated segmentation | Live cell images Time-lapse microscopy | AMTI Bayesian classifier | Accuracy | 63% |
| Bettauer 2022 [47] | *C. albicans* detection using morphologies | *C. albicans* | 1214 images (216/ 94/ -) | Manual labeling | Detection Classification | *NA* | Microscopic images | FCOS | Accuracy<br>Sensitivity<br>Precision<br>F1-score | 81.8%<br>82.4%<br>66.5%<br>73.7% |

| Author/ Year | Objective | Type of Species | Data Set (Training, Validation, Testing) | Eligibility criteria (if available) Labeling procedure | Machine Learning Task | Pre-processing | Type of Dataset | Model | Performance measured | Outcomes |
|---|---|---|---|---|---|---|---|---|---|---|
| Jamka 2021 [48] | Microorganism detection in cosmetic samples | *Bacillus subtilis, C. albicans, C. dubliniensis, C. glabrata, C. tropicalis, E. coli, Enterococcus faecalis, Klebsiella pneumoniae, Listeria monocytogenes, Morganella morganii, P. aeruginosa, P. mirabilis, P. vulgaris, Rhodococcus equi, S. agalactiae, S. aureus, S. epidermidis, Serratia marcescens, Salmonella typhimurium* | *NA* | *NA* | Detection Classification | *NA* | Microscopic images | CNN | Accuracy | 97% |
| Zawadzki 2021 [37] | Microorganism detection | *C. albicans, E. coli, P. aeruginosa, S. aureus* | 2,271 pairs of images (1816/ -/ 455) (for cell detection) 2,000 images (7:3:-) (for cell classification) | Gram staining and modifications | Detection Classification | Manual cell segmentation Images were resized, cut, and cropped. | Microscopic images | CNN DNN | Accuracy | 97% |

**Abbreviations:** AMIT: Algorithm for Migration and Interaction Tracking; CNN: Convolutional Neural Network; DNN: Deep, Neural Network; C. albicans: Candida albicans; C. dubliniensis: Candida dubliniensis; C. glabrata: Candida glabrata; C. tropicalis: Candida tropicalis; E. coli: Escherichia coli; FCOS: Fully Convolutional One-stage Object Detector; MRSA: Methicillin-resistant Staphylococcus aureus; P. aeruginosa: Pseudomonas aeruginosa; P. mirabilis: Proteus mirabilis; P. vulgaris: Proteus vulgaris; RNN: Recurrent Neural Network; S. agalactiae: Streptococcus agalactiae; S. aureus: Staphylococcus aureus; S. epidermidis: Staphylococcus epidermidis

(CNNs); including architectures like VGG16, ResNet50, EfficientNet, and fully convolutional one-stage object detectors (FCOS). Preprocessing steps often included image resizing, enhancement, and data augmentation.

#### Performance of Video-Based AI Models

Two studies utilized time-lapse microscopy videos to analyze neutrophil interactions with *Candida* species [46, 49]. The models used included CNNs, recurrent neural networks (specifically GRUs), and transformer-based architectures. Their performance varied depending on whether the input consisted of video sequences or static time-lapse frames and classification accuracy ranged from 96.2% to 100%, while sensitivity varied between 73% and 83%.

#### Performance of VOC-Based AI Models

One study used VOC time-series data captured from Petri dish cultures to identify *C. albicans* among other *Candida* species [1]. This study utilized time-series classification models, including KNN, InceptionTime, and HIVE-COTE ensembles. The accuracy ranged from 52.5% to 97.46%, sensitivity from 42.66% to 97.81%, specificity from 90.16% to 99.51%, precision from 39.47% to 97.54% and F1 score from 40.24% to 97.6%.

#### Meta-Analysis Feasibility

The execution of a meta-analysis was unfeasible owing to significant heterogeneity within datasets and the varied AI models employed. Moreover, a deficiency in reported essential metrics, such as true positive, true negative, false positive and false negative rates, further hindered the feasibility of conducting a comprehensive meta-analysis.

#### Risk of Bias Assessment

Figure 3 illustrates the outcomes of the risk of bias evaluations and applicability assessments conducted using QUADAS-2. Most studies showed low bias in flow and timing (86%). In the index test domain, 14%

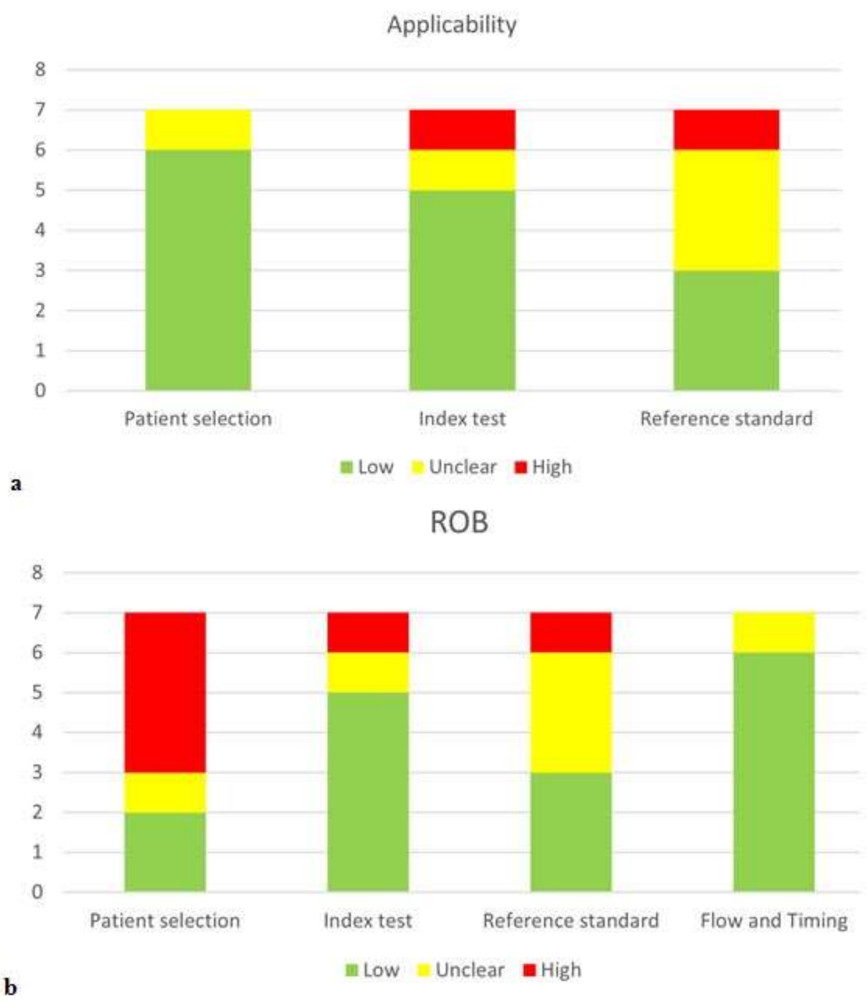

**Figure 3:** The outcome of the quality assessment of reviewed studies using the QUADAS-2 tool; **a:** The assessment of capability; **b:** The risk of bias assessment had high bias, 14% were unclear, and 72% showed low bias. However, patient selection mostly had a high bias (57%). Regarding the reference standard, 43% had a low bias, 14% had a high bias, and 43% were unclear.

## Discussion

Utilizing supplementary diagnostic methodologies can enhance the timely identification of fungal infections, consequently influencing treatment strategies and patient survival outcomes. Our review examined seven studies employing AI methodologies to identify and categorize *C. albicans*. Despite notable heterogeneity across the body of evidence, the AI models showcased considerable sensitivity, specificity, and accuracy across different study designs and reporting methods.

AI and ML are increasingly being integrated into medical imaging to support disease diagnosis, biomarker detection, prognosis prediction, and tissue classification [14, 23, 50]. This trend extends to the identification of fungal pathogens, where AI can process complex visual datasets with enhanced efficiency and consistency [51-56].

In this review, five of the included studies used microscopic images for the detection of *C. albicans* [14, 37, 46-48]. Two main AI approaches were identified: direct and indirect visual detection. The direct approach involves analyzing stained yeast images using ML models, whereas the indirect approach focuses on tracking immune cell behavior and morphological changes over time to infer infection presence [46].

Among the direct methods, Shankarnarayan *et al.* [14], Bettauer *et al.* [47], Jamka *et al.* [48], and Zawadzki *et al.* [37] applied CNNs to stained microscopic images of *C. albicans*. Shankarnarayan *et al.* [14] evaluated several CNN architectures and found that data augmentation significantly enhanced model performance. Their results revealed that data augmentation significantly improved model generalization and performance.

Specifically, the custom CNN model, when trained on augmented data, achieved a training accuracy of 85.4% and a validation accuracy of 83.9%. Nevertheless, this model showed a relatively low precision and recall, indicating room for improvement. Furthermore, the ResNet50 model, trained with data augmentation, demonstrated promising results, correctly predicting *C. albicans* raw images with 100.0% accuracy. Nevertheless, its performance varied across other *Candida* species, with accuracy ranging from 0.5% to 18.0%. The InceptionV3 model, also trained with data that were augmented, achieved 92.4% training accuracy and 78.7% validation accuracy. While this model accurately classified the majority of *C. albicans* images, it struggled with identifying *C. auris* and *C. haemulonii*. Notably, the InceptionV3 model outperformed other CNN models in classifying *Candida* species, indicating its potential for clinical applications.

Similarly, Jamka *et al.* [48] reported that InceptionV3 excelled at recognizing *C. albicans* but faced challenges in distinguishing morphologically similar species such as *C. glabrata* and *C. haemulonii*. Interestingly, the model's ability to predict different Candida species varied based on the type of image dataset used. For instance, the model achieved higher accuracy in identifying budding cells compared to single cells, suggesting that morphological features play a crucial role in classification. Moreover, the study highlighted the importance of dataset size and composition in ML model performance, with InceptionV3 demonstrating superior performance compared to other models [48].

Zawadzki *et al.* [37] corroborated these findings, emphasizing the efficacy of the InceptionV3 model in accurately classifying *Candida* species from microscopic images. They noted that while other CNN models, such as VGG16 and EfficientNetB0, yielded lower accuracies, InceptionV3 consistently outperformed them. The study underscored the challenges posed by morphological similarities among Candida species, necessitating further exploration of image features to improve classification accuracy [37].

In another study conducted by Bettauer *et al.* [47], Candescence, a deep learning-based tool designed for recognizing different morphologies of *C. albicans* from microscopy images was presented. Utilizing an FCOS trained with transfer learning, Candescence identifies and classifies nine *C. albicans* morphologies, including yeast white, opaque, shmoo, and hyphae. The system underwent iterative refinement, including a structured learning approach and a grid search for optimal hyperparameters. Despite challenges like overlapping objects and subtle morphological differences, Candescence achieved high accuracy in object detection and classification (an F1 score of approximately 73.7% with a recall of 82.4% and a precision of 66.5%). That highlighted the potential of this DL model in the identification of *Candida* species.

The indirect approach of detecting *C. albicans* technique was utilized in two studies [46, 49]. Sarkar *et al.* [46] and Belyaev *et al.* [49] tracked the movement and shape dynamics of neutrophils over time to identify the presence of *candida* species. Sarkar *et al.* [46] found that by analyzing individual microscopy frames, the CNN model was able to achieve 100% accuracy in identifying blood samples that were pathogen-free and distinguishing between *C. albicans* and *C. glabrata*.

Moreover, according to Belyaev *et al.* [49], they were able to achieve test accuracies of over 75% in distinguishing between *C. albicans* and *C. glabrata* infection scenarios, and perfect accuracy in identifying pathogen-free samples by using an ML-supported approach to time-lapse microscopy data.

Based on the studies conducted, both direct and indirect techniques can be utilized to identify and categorize *C. albicans* infections accurately. However, they each have distinct advantages and limitations that should be considered when choosing an appropriate method for microbiological control. Analyzing the microscopic images of microorganisms offers advantages like visual confirmation of their presence and accurate identification of microbial species based on their morphological characteristics. It allows researchers to evaluate the viability and quantity of microorganisms’ cells present in a sample, which is essential for the microbiological control of cosmetic products. Moreover, direct imaging methods can be customized as per specific staining techniques, such as gram staining, to enable the simultaneous evaluation of multiple microbial species like bacteria and yeasts, in a single microscopic preparation. This comprehensive analysis makes microbiological testing more efficient and accurate, ensuring regulatory compliance [14, 37, 47-48].

On the other hand, the indirect approach relies on the host immune response to detect Candida's presence, providing several advantages. One of its key benefits is its potential for high-throughput screening, where automated image analysis algorithms can rapidly assess immune cell behavior. This makes the indirect approach ideal for large-scale screening of cosmetic products, enabling the detection of Candida infections in various samples more efficiently. Additionally, this approach offers insights into the dynamic interactions between microorganisms and host immune cells, providing a deeper understanding of the mechanisms underlying fungal infections by focusing on host responses rather than direct pathogen detection [46, 49].

However, both approaches have certain limitations, as well. Directly imaging and analyzing microorganisms requires specialized equipment and expertise in microscopy and image analysis. These methods can be expensive and may not be readily available in all settings. Furthermore, the sample preparation and staining procedures required for direct imaging are time-consuming and labor-intensive, which makes it challenging to scale for large-scale screening. On the other hand, analyzing host immune responses also has its limitations. One notable challenge arises from the intricate interpretation of immune cell behavior, which exhibits considerable variation across individuals and can be affected by factors such as age, the overall condition of health, and genetic background. Due to this variability, it can be difficult to draw definitive conclusions about Candida infections based solely on immune cell phenotypes. In addition, analyzing time-lapse microscopy used in analyzing immune cell behavior can be challenging due to the time-intensive nature of the pre-processing steps required for video classification [14, 37, 46-49].

Detecting microorganisms can be done through VOCs. VOCs refer to a diverse array of molecules typically hydrophobic and based on carbon atoms, originating from the metabolic activities of fungi and bacteria, encompassing both their primary and secondary metabolic processes. These compounds are volatile, allowing them to easily disperse through the air and travel long distances [57]. The methods to detect VOCs include gas chromatography-mass spectrometry, solid phase microextraction, simultaneous distillation extraction, and selected ion flow tube mass spectrometry [58-62]. Another method is the electronic nose (E-Nose), which uses AI to detect patterns of VOCs and categorize them. Similar to a biological nose, an E-Nose endeavors to recognize patterns within VOCs detected by its sensors. These sensor readings are then scrutinized and categorized by an AI model [1, 58]. E-Nose is applied in various domains, including food safety [63-65], agriculture [57, 66-70], and medical diagnosis [59, 61-62]. Limitations such as sensor stability, standardization, and reliability require refinement, and progress is being made through the integration of AI and ML [1].

In this review, one study utilized E-Nose and ML to detect *C. albicans* [1]. The results of the study conducted by Bastos *et al.* [1] demonstrated the effectiveness of the proposed approach in accurately identifying Candida species. The AI models, particularly Inception Time, achieved high accuracy rates, with most models surpassing 90% accuracy in the validation and testing phases. Notably, Inception Time exhibited an average accuracy of 97.70%, underscoring its potential as a reliable model used for classifying volatile compounds produced by Candida species.

The integration of E-Nose technology with AI algorithms offers several advantages in Candida detection. Firstly, the use of E-Nose enables rapid and non-invasive sample analysis, facilitating timely diagnosis. The portability and relatively low cost of E-Nose devices further enhance their utility in various healthcare settings. Moreover, the study highlights the importance of Time Series analysis in capturing temporal patterns of VOC emissions, which are critical for accurate species identification [1].

In general, AI-based detection of *C. albicans* is a rapidly evolving field with multiple viable approaches. Microscopic imaging enables high-resolution, species-specific identification, while VOC detection offers rapid and scalable diagnostics. Each method has strengths and limitations, and the choice of technique should be informed by context-specific requirements such as available infrastructure, desired throughput, and diagnostic precision.

Future work should focus on improving model generalizability, particularly in morphologically similar species, and exploring hybrid approaches that combine imaging, VOC analysis, and immune profiling. Additionally, standardizing data collection and evaluation protocols will be essential to facilitate clinical translation and regulatory approval.

It is important to contextualize these findings within the quality of the included studies. The risk of bias assessment revealed that 57% of studies had a high risk of bias in patient selection, primarily due to the use of laboratory-controlled datasets and limited information regarding inclusion criteria. This presents a significant limitation; as such datasets may not fully reflect the diversity and complexity of real-world clinical scenarios. Consequently, the performance metrics reported- though promising- may be subject to overestimation and lack of generalizability. So, more representative sampling methods, multi-center datasets, and clearer documentation of participant selection processes are recommended in future studies to improve the robustness and applicability of AI models in real-world settings.

## Conclusion

This systematic review found that AI-based methods demonstrate strong potential in detecting *C. albicans* infections across various modalities, including microscopic imaging and VOC analysis. Both direct and indirect AI approaches showed high accuracy, sensitivity, and specificity in the included studies. While each technique has its strengths and limitations, the overall findings support the feasibility of AI-assisted diagnostic tools in identifying *C. albicans*, with further validation needed for clinical implementation.

## Acknowledgments

None.

#### Funding sources

No funding was received for this research.

## Conflict of Interest

The authors declare that they have no known competing financial interests or personal relationships that could have appeared to influence the work reported in this paper.
